# Comparing the Impact of Adolescent Dating Violence and Bullying on Anxiety Among LGBTQ+ and Heterosexual-Cisgender Adolescents in Vermont

**DOI:** 10.7759/cureus.102668

**Published:** 2026-01-30

**Authors:** Keelan Boisvert, Chanc VanWinkle Orzell, Madeleine Colton, Kylie Williams, Rebecca Brady, Elizabeth Woods, Elzerie De Jager, Thomas Delaney

**Affiliations:** 1 Department of Public Health, Larner College of Medicine, The University of Vermont, Burlington, USA; 2 Department of Medicine, Larner College of Medicine, The University of Vermont, Burlington, USA

**Keywords:** adolescents, anxiety, bullying, dating violence, lgbtq+, mental health, vermont

## Abstract

Objective

To examine how emotionally controlling behavior (ECB), adolescent dating violence (ADV), and bullying influence anxiety among LGBTQ+ and heterosexual-cisgender adolescents in Vermont.

Methods

A cross-sectional analysis of the 2021 Vermont Youth Behavioral Risk Survey (YRBS) was conducted among dating students (N=9007). Variables included sexual/gender identity, anxiety, ECB, physical dating violence, and bullying. Descriptive statistics and stratified binomial logistic regression were used, adjusting for demographics.

Results

Anxiety was reported by 68.7% of LGBTQ+ versus 30.2% of heterosexual-cisgender youth (p< 0.001). ECB and bullying significantly increased anxiety in both groups. For LGBTQ+ youth, ECB raised Odds (1-3 times, OR=2.1; 4+ times, OR=3.5), as did bullying (OR=2.1). Heterosexual-cisgender youth showed stronger associations (ECB Odds: 1-3 times OR=2.3, 4+ times OR=5.4; bullying OR=2.8). Physical dating violence was associated with anxiety for heterosexual-cisgender (OR: 1.4) but not LGBTQ+ youth (OR: 1.1).

Conclusions

ECB and bullying are strongly associated with anxiety, underscoring the need for targeted interventions addressing emotional abuse and bullying.

## Introduction

Anxiety is the most prevalent emotional disorder among adolescents, impacting academic success, social relationships, and physical and mental health [1]. Adolescence is a time for building social relationships, establishing healthy behaviors, and exploring identity. Lesbian, gay, bisexual, other sexual orientation, transgender, questioning, or unsure if they are transgender (LGBTQ+) adolescents who may face discrimination, stigma, and exclusion are at higher risk for mental health conditions like anxiety [1]. In Vermont, LGBTQ+ adolescents experience anxiety at significantly higher rates than heterosexual-cisgender peers (62% vs. 27%) [2]. Adolescent sexual and gender minority groups are more vulnerable to victimization, such as in-person bullying, online bullying, and adolescent dating violence (ADV), perpetuating disproportionate health outcomes [3,4].

Intimate partner violence (IPV), or ADV, involves harmful physical, sexual, or psychological behaviors between partners. ADV victimization is linked with internalizing symptoms, including anxiety and revictimization [5]. Among adolescents who have dated, non-heterosexual identity is strongly linked to partner victimization, with transgender and non-heterosexual individuals facing the greatest odds of ADV [3,6]. Vermont LGBTQ+ adolescents are more likely to report emotional and physical ADV victimization than heterosexual-cisgender peers [2].

In-person and online (cyberbullying) victimization has been shown to increase the likelihood of ADV victimization [3]. Described as repetitive, aggressive behavior perpetrated by one individual or group on a victim, bullying can depict power imbalances and increase the risk of anxiety [7]. Bullying is common nationally, with 19% of high schoolers bullied on school property and 16% cyberbullied in the past year [7]. LGBTQ+ youth report higher rates of bullying and cyberbullying victimization [8]. Vermont LGBTQ+ adolescents were more likely to be bullied in-person and online than heterosexual-cisgender peers [2].

Technology has become one of the primary ways for high school students to connect, with 77% using social media daily [8]. This shift highlights the need to address both cyberbullying and in-person bullying. This study aims to examine differences in the prevalence of emotional and physical adolescent dating violence (ADV), bullying, and anxiety between LGBTQ+ and heterosexual cisgender groups, with a primary objective of comparing the strengths of association between physical ADV, emotional ADV, and bullying with anxiety across these groups.

## Materials and methods

Study design

We conducted a cross-sectional study using data from the 2021 statewide Vermont Youth Behavioral Risk Survey (YRBS), restricting the analytic sample through convenience sampling to students who self-reported being in a dating or romantic relationship during the Fall 2021 survey period (September-December 2021), as the spring administration was cancelled due to COVID-19-related school modifications. This restriction was applied to ensure that measures of adolescent dating violence (ADV) reflected experiences within an active dating context, thereby improving the internal validity of analyses involving dating-related exposures. 

Inclusion and exclusion criteria

Inclusion criteria required participation in the 2021 YRBS, confirmation of dating during the survey period, and complete responses for all covariates, predictor variables, and the primary outcome (anxiety). Students were excluded if they did not report dating during the survey period or had missing responses to questions assessing dating experiences, emotionally controlling behavior, physical dating violence, bullying, anxiety, or key demographic covariates. As a result, findings may not be generalizable to adolescents who were not dating during the survey period or to broader youth populations and should be interpreted within the context of adolescents engaged in romantic relationships. 

Key variables

Key variables included LGBTQ+ status (LGBTQ+ vs. heterosexual cisgender), anxiety (never/rarely/sometimes vs. most of the time/always), emotionally controlling behavior (0 times, 1-3 times, 4+ times), physical dating violence (0 vs. 1+ times), and bullying (0 vs. 1+ times). Cyberbullying and in-person bullying were combined to create an inclusive measure capturing both traditional and emerging online forms of bullying; differences by bullying type were not examined separately in this analysis.

Statistical analysis

Descriptive analyses using chi-squared tests summarized demographics and key variables, followed by six stratified binomial logistic regression models estimating the odds of anxiety for LGBTQ+ youth (three models) and heterosexual-cisgender youth (three models), with each model analyzing one predictor (emotionally controlling behavior, physical dating violence, or bullying) while controlling for demographic covariates. Odds ratios (ORs) and 95% confidence intervals (CIs) were compared between LGBTQ+ and heterosexual-cisgender groups, and all analyses were conducted using SPSS version 29.

Ethical considerations

The project was conducted at The University of Vermont's Larner College of Medicine in a remote research format from August 2024 through April 2025. Because the study relied exclusively on pre-existing, de-identified survey data, it was not submitted for formal IRB review, as research faculty overseeing the project determined that it qualified as exempt from additional review. The University of Vermont Larner College of Medicine's Public Health Department Faculty were granted permission by the Vermont Department of Health to use the YRBS 2021 data set for analysis, and the full questionnaire is open access.

## Results

The analysis included 9007 Vermont adolescents who reported dating within the year prior to the 2021 YRBS survey (72.9% heterosexual-cisgender; 27.1% LGBTQ+). Cohort demographics included sex at birth, age, ethnicity, and rurality (Table 1). LGBTQ+ youth experienced higher rates of anxiety compared to their heterosexual-cisgender peers (most of the time or always: 68.7% vs. 30.2%, p< 0.001). Among LGBTQ+ students, 22% reported emotionally controlling behavior from a romantic partner 1-3 times, and 20.6% reported 4 or more times. For heterosexual-cisgender youth, 14.4% reported emotionally controlling behavior 1-3 times, and 10.6% reported 4 or more times. Physical dating violence was more prevalent among LGBTQ+ adolescents compared to their heterosexual-cisgender peers (9.4% and 7.4%, p=0.002); as was bullying (47.0% and 25.7%, respectively, p< 0.001).

**Table 1:** Demographics of the study population. p-value is calculated using Pearson’s Chi-square test with a statistical significance threshold of p=0.05.

Characteristics	Overall (n=9007)	LGBTQ+ (n=2440; 27.1%)	Heterosexual–cisgender (n=6567; 72.9%)	χ²	p-value
Age, n (%)				6.44	0.04
Less than 12–14	1252 (13.9)	371 (15.2)	881 (13.4)		
15–17	7048 (78.3)	1893 (77.6)	5155 (78.5)		
Older than 18	684 (7.6)	168 (6.9)	515 (7.8)		
Sex at birth, n (%)				864.81	<0.001
Male	4378 (48.6)	544 (22.3)	3834 (58.4)		
Female	4529 (50.3)	1807 (74.1)	2722 (41.4)		
Race, n (%)				29.01	<0.001
BIPOC	1429 (15.9)	468 (19.2)	961 (14.6)		
White	7509 (83.4)	1941 (79.6)	5567 (84.8)		
Rurality, n (%)				0.08	0.78
Urban	1876 (20.8)	513 (21.0)	1363 (20.8)		
Rural	7131 (79.2)	1927 (79.0)	5204 (79.2)		
Physical dating violence, n (%)				9.33	0.002
Yes	720 (8.0)	230 (9.4)	490 (7.5)		
No	8287 (92.0)	2210 (90.6)	6076 (92.5)		
Emotionally controlling behavior, n (%)				273.64	<0.001
0 times	6329 (70.3)	1402 (57.5)	4927 (75.0)		
1–3 times	1481 (16.4)	536 (22.0)	944 (14.4)		
≥4 times	1197 (13.3)	502 (20.6)	696 (10.6)		
Bullying, n (%)				374.35	<0.001
Yes	2835 (31.5)	1147 (47.0)	1688 (25.7)		
No	6172 (68.5)	1293 (53.0)	4879 (74.3)		
Anxiety, n (%)				1086.25	<0.001
Most of the time/always	3661 (40.6)	1676 (68.7)	1985 (30.2)		
Never/rarely/sometimes	5264 (58.4)	749 (30.9)	4515 (68.8)		

Exposure to emotionally controlling behavior by a dating partner 4 or more times had the strongest association with anxiety for both LGBTQ+ and heterosexual-cisgender youth. For LGBTQ+ adolescents, however, increased exposure from 1-3 times to 4 or more times was not significantly different (1-3 times: OR=2.1, 95% CI (1.6, 2.7), p<0.001; 4 or more times: OR=3.5, 95% CI (2.6, 4.6), p<0.001). Among heterosexual-cisgender adolescents, a dose-response was observed as exposure increased (1-3 times: OR=2.3, 95% CI (2.0, 2.7), p<0.001; 4 or more times: OR=5.4, 95% CI (4.5, 6.5), p<0.001).

Bullying increased the odds of anxiety for both LGBTQ+ and heterosexual cisgender adolescents (LGBTQ+: OR=2.1, 95% CI (1.8, 2.6), p<0.001; heterosexual cisgender: OR=2.8, 95% CI (2.4, 3.1), p<0.001; see Table 2). Physical dating violence was not significantly associated with anxiety among LGBTQ+ youth, which may reflect limited sample size, measurement sensitivity, or differential reporting of physical violence experiences, but did significantly increase the odds of anxiety among heterosexual cisgender youth (LGBTQ+: OR=1.1, 95% CI (0.8, 1.5), p=0.535; heterosexual cisgender: OR=1.4, 95% CI (1.1, 1.7), p<0.001).

**Table 2:** Stratified logistic regression analysis of adverse partner experiences among LGBTQ+ and heterosexual-cisgender youths who were dating within 12 months of taking the 2021 YRBS. Reported are Odds Ratios (OR) with 95% confidence intervals and p-values using a statistical significance threshold of p=0.05.

Cohort	Adverse interactions	Exposulre level	OR (95% CI)	p-value
LGBTQ+ cohort	Emotional controlling behavior	0 times	Reference	Reference
		1–3 times	2.1 (1.6, 2.7)	<0.001
		≥4 times	3.5 (2.6, 4.6)	<0.001
	Physical dating violence	0 times	Reference	Reference
		>1 time	1.1 (0.8, 1.5)	0.535
	Bullying	No	Reference	Reference
		Yes	2.1 (1.8, 2.6)	<0.001
Heterosexual–cisgender cohort	Emotional controlling behavior	0 times	Reference	Reference
		1–3 times	2.3 (2.0, 2.7)	<0.001
		≥4 times	5.4 (4.5, 6.5)	<0.001
	Physical dating violence	0 times	Reference	Reference
		>1 time	1.4 (1.1, 1.7)	0.001
	Bullying	No	Reference	Reference
		Yes	2.8 (2.4, 3.1)	<0.001

These exposures increased anxiety odds for all dating adolescents. The association was statistically significant among heterosexual cisgender youth but was not statistically significant among LGBTQ+ youth.

## Discussion

Previous studies have explored how anxiety and adverse experiences impact youth mental health [9,10]. Our study extends this work by focusing specifically on bullying and both physical and emotionally controlling dating violence, comparing their associations with anxiety among LGBTQ+ and heterosexual-cisgender adolescents. Bullying and ADV were strongly associated with anxiety in both groups, reinforcing the importance of continued research on these exposures among adolescents [9,10]. Emotionally controlling behavior from dating partners was also associated with anxiety, with a dose-response effect observed among heterosexual-cisgender youth, consistent with prior findings [9].

Our results highlight the need for continued research on diverse youth populations and the development of inclusive, evidence-based interventions. This aligns with prior studies identifying gaps in the literature on LGBTQ+ youth and mental health [11,12]. Our findings reinforce calls for more research on ADV and anxiety, particularly in LGBTQ+ youth [11,12]. Continued exploration of LGBTQ+ experiences across diverse settings is essential to inform interventions that address bullying, ADV, and emotional abuse. Research that centers on the lived experiences of LGBTQ+ youth can guide public health, clinical, and community strategies to improve mental well-being and promote health equity.

Limitations

Our study has several limitations. The sample was limited to Vermont, which may limit generalizability to other regions. Additionally, the cross-sectional and self-reported nature of the YRBS data introduces the potential for recall and response biases and limits our ability to infer causality. The ADV items measured frequency, but did not capture context, such as severity, support systems, or power dynamics.

## Conclusions

LGBTQ+ youth experienced higher levels of bullying and ADV and reported substantially higher prevalence of anxiety compared with heterosexual cisgender youth. Emotionally controlling behaviors and bullying were associated with increased odds of anxiety among adolescents who were dating, regardless of sexual orientation or gender identity. Emotional control emerged as the exposure most strongly associated with anxiety in both groups. A graded pattern of increasing anxiety with greater exposure to emotionally controlling behavior was observed among heterosexual cisgender youth, while elevated odds were present across exposure levels among LGBTQ+ youth. Bullying was also associated with anxiety in both cohorts. Physical dating violence was associated with anxiety among heterosexual cisgender adolescents but not among LGBTQ+ adolescents in this sample. Overall, these findings highlight the central role of non-physical interpersonal stressors in adolescent anxiety within dating and peer contexts
